# Correlation of cardiac magnetic resonance imaging and biochemical markers of myocardial injury in a multi-centre study: PROTECTION AMI CMR substudy

**DOI:** 10.1186/1532-429X-14-S1-O66

**Published:** 2012-02-01

**Authors:** Suchi Grover, Gregory Bell, Christine M Edwards, Saling Huang, Darryl P Leong, Lucas Jeorg, Per Lav Madsen, Cameron Bridgman, Sean K Leow, Adhiraj Chakrabarty, Joseph Selvanayagam

**Affiliations:** 1 Flinders Cardiac Imaging Research Centre, Flinders Medical Centre, Adelaide, SA, Australia; 2 Flinders University, Adelaide, SA, Australia; 3 KAI Pharmaceuticals, San Francisco, CA, USA

## Summary

The purpose of this study was to determine predictors of 90-day left ventricular function following acute ST-segment elevation myocardial infarction (STEMI) using variables from clinical presentation, biomarker testing, and cardiovascular magnetic resonance imaging (CMR).

## Background

Identifying patients with acute STEMI who experience adverse remodelling and develop left ventricular dysfunction 3 months post-MI is a priority for guiding subsequent therapy. To our knowledge, this is the first extensive multi-centre study utilizing CMR assessment in patients with anterior STEMI.

## Methods

Consecutive patients undergoing primary PCI for anterior MI as part of Inhibition of Delta-protein Kinase C for the Reduction of Infarct Size in Acute Myocardial Infarction (PROTECTION-AMI) trial, were enrolled into the CMR sub-study. CMR was performed at baseline (day 3 to 5) and 3 months and used to evaluate infarct size (delayed hyperenhancement, DHE), myocardial salvage, microvascular obstruction (MVO), transmural extent of hyper-enhancement, regional and global left ventricle function. Myocardial oedema imaging was used to define the area at risk and subsequent calculation of myocardial salvage index (MSI). Two independent observers, blinded to functional and clinical data, performed the myocardial oedema and infarct size assessment separately. Biochemical markers including creatine kinase area under the curve (CK AUC), peak CK and troponin I (Tn I) were collected at specific time-points.

## Results

95 patients were enrolled in the sub-study and 85 completed the 3 month follow-up, across 24 centres worldwide. Patients were 63 ± 12 years of age, 77% male and median time of symptom onset to infusion of drug was 180 ± 90 minutes (q25 111, q50 170, q75 226). Assessment of infarct size and MSI was performed in 81/85 (95%) and 56/85(66%)of patients respectively. Median LVEF was 56% (45 to 63) at baseline and 60% (49 to 67) at 3 months (p<0.001). For every increase in one grade of DHE transmurality, the odds ratio of a segmental improvement in regional wall motion was almost halved (0.46, 95% confidence intervals 0.41 - 0.52, p < 0.001). There was moderate correlation with CK AUC and infarct size (IS) by CMR at baseline r = (0.66, p < 0.001). IS had moderate inverse correlation with 3 month EF (r = -0.52, p < 0.0001) and was favourable as compared to MSI (r = -0.39, p < 0.0004) or MVO (r = -0.3, p < 0.009). CK AUC, CK peak and Tn I peak all correlated favourably with LVEF at 3 months (r = -0.52, -0.53 and -0.56 respectively). In a multivariable model with biomarker and imaging variables, only baseline CMR infarct size independently predicted 90-day LVEF.

## Conclusions

This is the first report of a combined CMR protocol (including myocardial oedema imaging) in a multi-centre, multi-vendor setting. Although both IS and MSI were univariate predictors of late functional recovery, only baseline CMR infarct size independently predicted 90 day left ventricular function. Infarct size using the late gadolinium technique remains the most robust CMR tool to predict late LV systolic dysfunction following STEMI. Our findings confirm previous studies showing significant correlations between baseline biochemistry and acute CMR infarct size and between baseline biochemistry and late LVEF.

## Funding

The PROTECTION main and sub-study were funded by KAI-Pharmaceuticals and Bristol-Myers Squibb.

**Figure 1:**
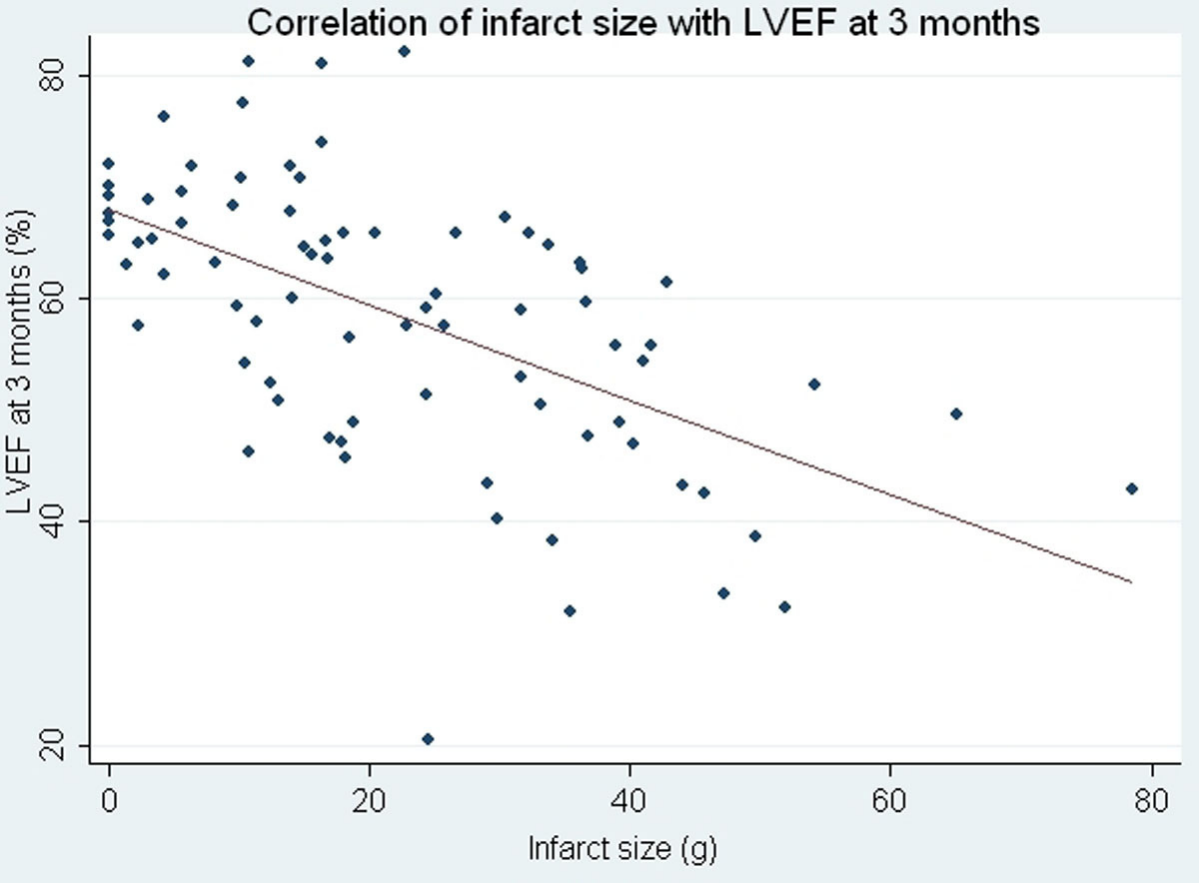
A displays relationship between between infarct size by CMR with LVEF at 3 months.

**Figure 2:**
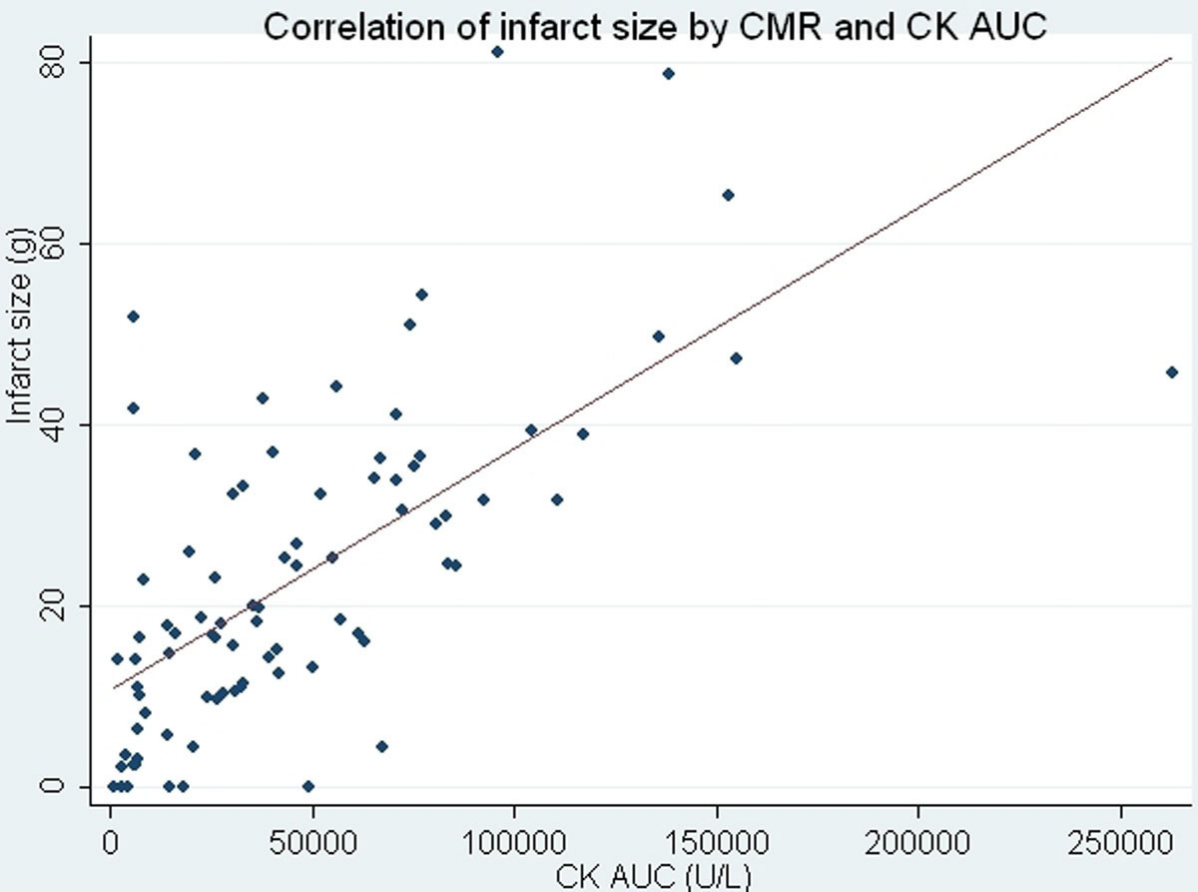
displays relationship between CK AUC and infarct size by CMR at baseline.

